# Encephalopathy in Preterm Infants: Advances in Neuroprotection With Caffeine

**DOI:** 10.3389/fped.2021.724161

**Published:** 2021-10-01

**Authors:** Liu Yang, Xuefei Yu, Yajun Zhang, Na Liu, Xindong Xue, Jianhua Fu

**Affiliations:** ^1^Department of Pediatrics, Shengjing Hospital of China Medical University, Shenyang, China; ^2^Department of Pediatrics, The Second Hospital of Dalian Medical University, Dalian, China; ^3^Department of Anesthesiology, Dalian Municipal Maternal and Child Health Care Hospital, Dalian, China

**Keywords:** caffeine, premature infants, brain damage, encephalopathy, neurological

## Abstract

With the improvement in neonatal rescue technology, the survival rate of critically ill preterm infants has substantially increased; however, the incidence of brain injury and sequelae in surviving preterm infants has concomitantly increased. Although the etiology and pathogenesis of preterm brain injury, and its prevention and treatment have been investigated in recent years, powerful and effective neuroprotective strategies are lacking. Caffeine is an emerging neuroprotective drug, and its benefits have been widely recognized; however, its effects depend on the dose of caffeine administered, the neurodevelopmental stage at the time of administration, and the duration of exposure. The main mechanisms of caffeine involve adenosine receptor antagonism, phosphodiesterase inhibition, calcium ion activation, and γ-aminobutyric acid receptor antagonism. Studies have shown that there are both direct and indirect beneficial effects of caffeine on the immature brain. Accordingly, this article briefly reviews the pharmacological characteristics of caffeine, its mechanism of action in the context of encephalopathy in premature infants, and its use in the neuroprotection of encephalopathy in this patient population.

## Introduction

With the improvement in rescue technology, the survival rate of critically ill newborns has substantially increased. It is estimated that 9 million (60%) of the 15 million premature babies born each year will experience lifelong physical or neurological disabilities (1–3). Premature infants have short gestational age and relative immature development of various organs compared to full-term infants, and are thus more susceptible to unfavorable perinatal environments, which may lead to the damage of some organs, especially the brain. In recent years, some scholars have proposed that brain damage related to premature birth can be collectively referred to as “encephalopathy of prematurity (EP)” (4). The term EP signifies that brain injury in premature infants is not limited to brain white matter, but also involves the gray matter and cerebellum. The main pathological features of EP include myelin sheath reduction, oligodendrocyte-maturation disorder, axonopathy, and neuroinflammation; moreover, fractional anisotropy and cortical volume appear to be reduced on magnetic resonance imaging. This plays a role in the impairments that are frequently seen in the very preterm population (in cognition, language, behavior, and the motor system) (4, 5). Although the etiology and pathogenesis of EP and its prevention and treatment have been important topics of discussion in recent years, powerful and effective neuroprotective strategies have so far not been established. Animal experiments have confirmed that drugs such as magnesium sulfate and melatonin or methods such as recombinant erythropoietin administration and breastfeeding play a certain role in the prevention and treatment of brain injury in premature infants (6, 7). Additionally, caffeine as a potential treatment has also been suggested, which it is considered a promising drug for improving perinatal brain-injury outcomes (8).

As a methylxanthine drug, caffeine has been used in neonatal intensive care units for the treatment of neonatal apnea for over 30 years (9, 10). Clinical studies have shown that caffeine has neuroprotective effects in premature infants by alleviating hypoxia-induced white matter damage, and improving ventilation function and brain self-regulation (11, 12). In addition, caffeine has been shown to reduce the apoptosis of developing brain neurons, ventricular enlargement, and white matter loss caused by hypoxia (13–15) and myelination disorders (16). However, few studies have examined the molecular mechanisms implicated in the neuroprotective effect conferred by caffeine treatment. This article reviews the pharmacological characteristics of caffeine, its mechanism of action in the context of encephalopathy in premature infants, and its use in EP.

## Pharmacological Characteristics of Caffeine

### Pharmacological Effects of Caffeine

Caffeine (1,3,7-trimethylxanthine, C_8_H_10_N_4_O_2_) is a methylxanthine drug. When consumed, it is distributed throughout the body, including the brain, heart, blood vessels, and kidneys. In the brain, it stimulates the respiratory center, increases respiratory frequency and tidal volume, improves pulmonary blood flow, increases the body's sensitivity to carbon dioxide, enhances diaphragm function, and stimulates the respiratory center (17–19); it also acts as a central nervous system stimulant and hypnotic. Previous studies have shown that caffeine antagonizes the adenosine receptor (ARs) and mainly functions by non-specifically antagonizing the adenosine A 1 receptor (A1R) and adenosine A2a receptor (A2aR) (20). Blockade of A1R and A2a indirectly affects other neurotransmitters in the brain, such as dopamine, serotonin, norepinephrine, acetylcholine, and γ-aminobutyric acid (GABA) (17). In addition, caffeine can stimulate the heart muscle and increase the heart rate, cardiac output, and mean arterial blood pressure by promoting the release of catecholamines. In blood vessels, caffeine can expand vascular smooth muscle cells by increasing the nitric oxide concentration (20, 21). In the kidneys, caffeine can increase the glomerular filtration rate and produce diuretic effects by antagonizing adenosine A1Rs (22).

### Route of Administration, Pharmacokinetics, Dosage, and Time-Course of Caffeine

#### Route of Administration and Pharmacokinetics of Caffeine

Caffeine is medically available as caffeine citrate, and the clinically commonly used routes of administration are oral and intravenous. Previously, caffeine was also administered as an intramuscular injection in the form of caffeine benzoate. However, because this impairs the ability of newborn albumin to bind bilirubin, this formula has become unpopular (23). In adults, the time for an oral dose to reach its peak is 30 min to 2 h (24). Within 45 min, 99% of caffeine will be absorbed, most of which by the small intestine (25). Caffeine reversibly binds to plasma proteins, and caffeine citrate is equivalent to 50% of caffeine. Therefore, a load of caffeine citrate will produce a relatively predictable serum concentration (26). In most preterm infants, the plasma caffeine concentration is maintained at 5–20 mg/ml after a loading dose of 20 mg/kg and a maintenance dose of 5 mg/kg/day (18). Caffeine, being highly fat-soluble, can enter the cerebrospinal fluid (CSF) through the blood-brain barrier and quickly distribute into the brain tissue. The drug concentration in the neonatal CSF is similar to that in the plasma. However, the liver enzyme system and kidney function of premature infants are immature, and compared with adults, metabolism in the body is very limited and the clearance rate is slow; most active drugs are excreted in the urine. In premature infants, the primary metabolic pathway of caffeine involves the process of 7-N-demethylation.

#### Dosage of Caffeine

##### Treatment of Apnea Using Caffeine

Early administration of high-dose caffeine is recommended for the treatment of apnea of prematurity (AOP). Experimental research trials testing caffeine for the treatment of AOP (caffeine for apnea of prematurity; CAP) have revealed that caffeine treatment started within 3 days after birth has the most significant effect (27). A retrospective study showed that, compared with the use of caffeine after 3 days, its use within 3 days is associated with a reduction in the incidence of bronchopulmonary dysplasia and improved prognosis (28, 29). Although some studies have shown that the use of caffeine at different times in the early and late stages has no effect on mortality, there are indeed studies confirming that the reduced incidence of AOP is related to the early use of caffeine (30–33). In short, it is beneficial to start caffeine treatment within 3 days after birth. The dose of caffeine citrate used for the treatment of AOP, as prescribed by the U.S. Food and Drug Administration, is a 20 mg/kg load and 5 mg/kg/day maintenance dose; this standard-dose concentration allows more than 70% of newborns to reach therapeutic levels of 8–20 mg/L (23, 25). However, in the past, many hospitals have used maintenance doses ranging from 5 to 10 mg/kg.

Many studies have shown that higher doses of caffeine are more effective than the standard maintenance dose, and no increase in side effects has been reported (34, 35). A randomized controlled trial showed that different maintenance doses of caffeine citrate (3, 15, 30 mg/kg) showed no difference in the extubation failure rate among groups of newborns with a gestational age of <32 weeks. However, the incidence of AOP in the two high-dose groups was significantly reduced compared with the incidence in the lowest-dose group (35). Another study compared neonates with a gestational age of <28 weeks at birth who were administered different doses of caffeine citrate (20 mg/kg/day vs. 5 mg/kg/day) during extubation, and the failure rate in the high-dose group was significantly reduced [15 vs. 29.8%; relative risk 0.51 (0.31–0.85)] (25). Subsequently, a 1–2-year follow-up of these cohort studies revealed that these neonates did not show significant differences in neurodevelopment (36).

A recent trial compared high-dose (loading 40 mg/kg, maintenance 20 mg/kg/day) and standard-dose (loading 20 mg/kg, maintenance 10 mg/kg/day) caffeine citrate administration. The failure rate of extubation, frequency of apnea, and number of days of apnea in the high-dose group were significantly lower than those in the standard-dose group (34). In addition, studies have shown that when the loading dose of caffeine citrate reaches 50 mg/kg and the maintenance dose reaches 20 mg/kg/day, it is more effective in reducing apnea episodes and promoting extubation (37). A recent systematic review and meta-analysis of caffeine in the treatment of AOP showed that high maintenance doses of caffeine citrate were more effective and safer than low maintenance doses during treatment (33). The positive results of these tests, coupled with the minimal side effects of the high-dose regimen, have led to the routine use of caffeine citrate maintenance doses of up to 20 mg/kg/day.

##### Neuroprotection of Encephalopathy Using Caffeine

There is currently no uniform caffeine dosage or administration timing for the neuroprotection of EP. Several long-term randomized controlled trials have studied the effects of caffeine administration timing on the nervous system of premature infants. A study of very premature infants with a gestational age of <29 weeks showed that the early caffeine group (within 2 h, 20 mg/kg) in the early postnatal period showed greater circulatory improvement (i.e., improved blood pressure and systemic blood flow) than the conventional caffeine group (within 12 h, 20 mg/kg) (38). The latest study suggested that early caffeine treatment is associated with better neurodevelopmental results (39). A study compared preterm infants with a gestational age of <29 weeks who were treated with caffeine early (medication received within 2 days from birth) and late (medication received after 2 days of birth). The incidences of cerebral palsy hearing damage and a Bayley scale cognitive score for infant development of <85 were decreased in the early-caffeine group (39) compared to the late-caffeine group. These data indicate that early use of caffeine could lead to more beneficial neurodevelopmental outcomes.

In premature infants with a gestational age of <30 weeks, a study compared neonates who received high-dose caffeine citrate (80 mg/kg, for longer than 36 h) and the standard dose of caffeine citrate (30 mg/kg, for longer than 36 h). The incidence of cerebellar hemorrhage (CBH) in the high-dose group increased (36 vs. 10%, *p* = 0.03), as did the incidence of epilepsy and neurobehavioral abnormalities at full-term (corrected for gestational age) (40, 41). However, a more recent study showed no difference in the incidence of CBH between high-dose (80 mg/kg/d) and standard-dose (20 mg/kg/d) caffeine citrate administration in very preterm infants with gestational age <28 weeks. In addition, there was no difference between the two groups at the age of 2 years according to the Neurosensory Motor Development Assessment and Bayley Infant Development Scale III (Bayley III) (42). Although these results have allayed concerns regarding the use of high-dose caffeine in early preterm infants, it is notable that no study has reported the advantages and disadvantages of using high-dose caffeine in preterm infants with small gestational age. Therefore, early high-dose caffeine and its effects on brain injury, such as intraventricular hemorrhage and on long-term neurodevelopmental outcome, need to be determined on a larger scale.

#### The Time-Course of Caffeine

Discontinuation of conventional caffeine treatment is usually after the apnea symptoms disappear, that is, at a corrected gestational age of 33–35 weeks. The median time to the last dose in the CAP trial (9) was 34.4 weeks corrected gestational age (IQR, 33.0–35.9). Although intermittent hypoxia persists after caffeine treatment is stopped, caffeine reduces the degree of intermittent hypoxia (43, 44). A recent study showed that (45) premature infants with a birth weight ≤1,250 g should be treated with caffeine within 1 week after birth, while the length of treatment had no effect on the long-term neurodevelopmental outcome at 3 years of age. As there is no clinical evidence that caffeine can continuously improve intermittent hypoxia, acute injury, or long-term prognosis, the current routine clinical withdrawal time remains at 33–35 weeks corrected gestational age (18).

In conclusion, for the neuroprotection of EP, early use of caffeine could lead to more beneficial neurodevelopmental results. However, despite the short-term benefits of high-dose caffeine in the prevention and treatment of apnea, the long-term efficacy and safety of these regimens for the nervous system have not been evaluated. The current standard dose of caffeine is considered safe and reliable. In addition, the time-course of caffeine can only be increased after it is proven that caffeine can improve the long-term prognosis and that this is not associated with adverse reactions.

## Neuroprotective Mechanism of Caffeine

The drug action of caffeine involves three basic mechanisms: it is an AR antagonist, a phosphodiesterase inhibitor, and an active intracellular calcium mobilizer (46). In addition, it can also interfere with GABA-A receptors, change GABA transport (47, 48), and inhibit the production and activation of prostaglandins (19). Because caffeine has a series of molecular targets in the central nervous system, it is difficult to determine its exact molecular and cellular mechanisms in preventing and treating encephalopathy in premature infants.

### Neuroprotective Mechanism of Caffeine and the Expression and Regulation of Adenosine Receptors

Adenosine is a neuromodulator of which metabolism depends on the synthesis, release, and decomposition of adenosine triphosphate. Known as adenine nucleoside, it exists in all cells and is a component of nucleic acid and energy-carrying molecules (49, 50). In brain tissue, adenosine is mainly produced by neurons and plays an important role in the development of brain injury in immature infants, such as periventricular white matter injury (51). Following increased tissue activity, hypoxia, or ischemia, the adenosine levels in brain tissue increase, triggering the activation of ARs (52, 53). All ARs are G-protein coupled receptors, including A1R, A2aR, A2bR, and A3R. A1R and A2aR may be the main targets of caffeine. The A1R receptors inhibit cyclic adenosine monophosphate (cAMP) production, whereas A2aR receptors stimulate cAMP production by adenylate cyclase. The distribution of A1R mRNA in mature brain tissue is the most extensive, mainly concentrated in the hippocampus, cerebellum, and cerebral cortex. The content of A2aR mRNA in the striatum and globus pallidus is high, and it is also found in astrocytes, microglia, and blood vessels. A3R is distributed in cortical nerve endings, hippocampal cells, astrocytes, and microglia (54). Caffeine and adenosine have similar molecular structures; therefore, caffeine plays a role in all types of ARs. When caffeine acts as an AR antagonist, it can compete against ARs, which has multiple potential effects on the developing brain (33, 51). A3R and a2bR need high concentrations of adenosine to activate, while A1R and A2aR can be activated at lower concentrations. As such, most research has focused on A1R and A2aR.

#### Expression and Regulation of A1R

Caffeine's inhibition of A1R has opposite effects on embryonic and newborn brains. In the embryonic stage, inhibition of A1R renders cells more susceptible to hypoxia, which adversely affects fetal neurodevelopment and long-term behavior (55). However, postnatal inhibition of A1R in neonatal brains exerts a neuroprotective effect against hypoxic-ischemic white matter damage (16, 51). Activation of A1R results in increased adenosine levels in the body, causing damage to the developing brain (56). Therefore, postnatal application of caffeine can improve the development of neonatal white matter by antagonizing A1R (57), which is considered beneficial for the long-term neurodevelopment of premature infants. Studies have shown that in perinatal brain injury models, overactivation of A1R can lead to premature white matter damage by changing the development of oligodendrocytes (58), while caffeine antagonizing A1R can reduce ventricular enlargement and white matter loss caused by hypoxia, increase the proportion of immature oligodendrocytes, and protect against periventricular white matter injury and hypoxic-ischemic encephalopathy (13). Recent studies have shown that *in vitro* (59), the expression of A1R is significantly upregulated and the expression of transcription factors of oligodendrocytes is significantly decreased under hypoxia. Caffeine can promote the differentiation and maturation of oligodendrocytes and the expression of myelin-related proteins *in vitro* by antagonizing A1R.

**Figure 1:**
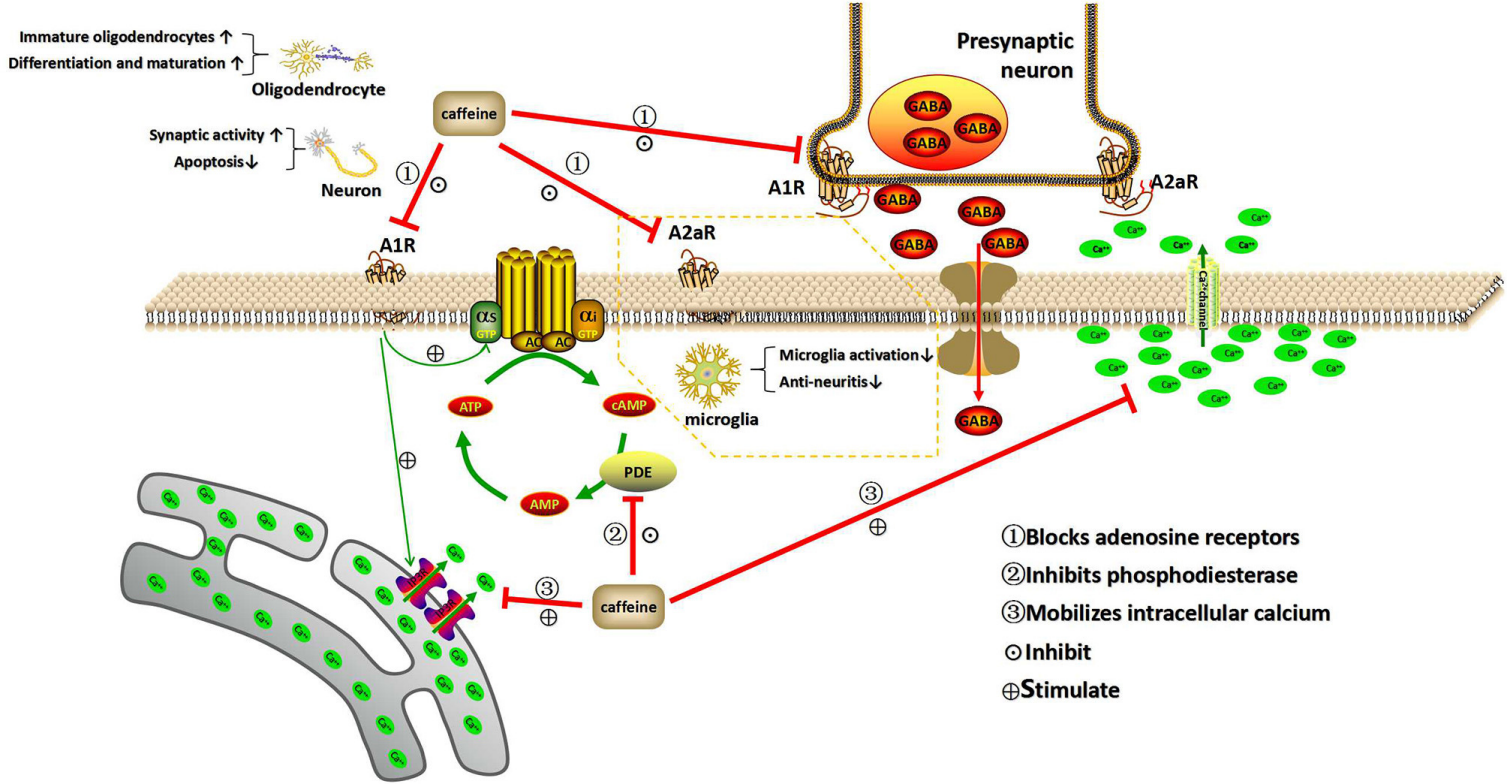
Mechanism of action of caffeine. This illustration depicts the three basic mechanisms of caffeine within the central nervous system. Caffeine acts as an adenosine receptor antagonist, phosphodiesterase inhibitor, and intracellular calcium mobilizer. A1R and A2aR can be activated at low concentrations of adenosine, while A3R and A2bR require higher concentrations of adenosine to become activated. Therefore, A1R and A2aR are the main targets of caffeine in the nervous system. As an adenosine receptor blocker, caffeine can reduce neuronal apoptosis and weaken the synaptic activity inhibited by hypoxia, increase the proportion of immature oligodendrocytes after hypoxia, and promote the differentiation and maturation of oligodendrocytes. It also blocks A2aR and inhibits microglia activation. Caffeine is an inhibitor of phosphodiesterase, which can prevent the breakdown of cAMP. As an internal calcium mobilizer, caffeine can be combined with calcium channels to release calcium from cells, while inhibiting voltage-sensitive calcium channels and neurotransmission, and mobilize the transmission of intracellular calcium (Ca^2+^) through the inositol triphosphate receptor (IP_3_R). In addition, caffeine can interfere with GABA transport by blocking ARs and can affect the release of a variety of neurotransmitters. A1R, A2aR, A2bR, and A3R, adenosine receptors; cAMP, cyclic adenosine monophosphate; AC, adenylate cyclase; PDE, phosphodiesterase; AMP, adenosine monophosphate; GTP, guanosine triphosphate; IP_3_R, inositol triphosphate receptor; GABA, γ-aminobutyric acid.

The above-described experiments have confirmed that caffeine antagonizing A1R may reverse hypoxia-induced white matter injury in premature infants. In addition, the neuroprotective effect of caffeine against A1R may be related to its anti-apoptotic effect. Adenosine synthesis and decomposition are unbalanced during hypoxia-ischemia. When the energy demand exceeds the energy supply, the concentration of extracellular adenosine increases, and brain cells are at a risk of death. By blocking A1R in cord blood monocytes, caffeine increases the production of cAMP, reduces the production of pre-transcriptional TNF-α (60), inhibits the inflammatory response, and reduces cell apoptosis. Inhibition of TNF-α may be one of the mechanisms through which caffeine causes anti-hypoxic-ischemic brain injury-induced cell apoptosis. Studies have shown that the number of apoptotic cells in the hippocampus and parietal cortex of neonatal rats significantly decreased after caffeine treatment in a hypoxic-ischemic brain injury model through A1R antagonism (14). These data indicate that the neuroprotective effect of caffeine may also be related to its anti-apoptotic effect. Research by Li et al. (61) showed that the use of caffeine in the early postnatal period (P4-P7) of newborn rats can reduce CoCl_2_-induced cell death and inhibit the accumulation of HIF-1α in the nucleus by blocking A1R, thereby reducing the damage caused by hypoxia in developmental neurons. Additionally, as an A1R antagonist, caffeine can affect synaptic transmission in the hippocampus (62), and increase the release of neurotransmitters (63) (Figure 1).

#### Expression and Regulation of A2R

Previously, the ability of caffeine to induce central nervous system excitation was mainly attributed to its effect on A1R because the A1R receptor protein is expressed in the brainstem. In addition, adenosine-induced A1R activation was found to have an inhibitory effect on neurons (64). However, recent studies have shown that A2aR may also be involved. Caffeine-induced central nervous system arousal has been shown to be associated with A2aR (65). In rats, caffeine promotes wakefulness by inhibiting the expression of A2aR. When A2aR is removed from the nucleus accumbens or other A2aR positive regions of the basal ganglia, wakefulness is blocked. In addition, the activation of A2aR also contributes to the repair of ischemic tissue damage (66, 67). There may be a link between the effects of caffeine, adenosine levels, perinatal inflammation after delivery, and imbalance between pro-inflammatory and anti-inflammatory signaling in preterm infants (68). Studies have shown that the AR most relevant to neuroinflammation is A2aR (69). Although its expression in microglia is usually low, it significantly increases after brain injury. In microglia, activation of A2aR can promote the release of cytokines and changes in amoebic morphology (70). In contrast, A2aR antagonists can inhibit the activation of microglia (71, 72). As an A2aR antagonist, caffeine prevents this change, as well as neuroinflammation (72). This protective effect may be exerted through A2aR by regulating the activation of microglia and the secretion of brain-derived neurotrophic factor (71). Colella et al. (73) revealed that A2aR agonist CGS-21680 can cause increase in the level of the CD73 protein and in the expression of M1 (pro-inflammatory) cytokines (IL-1β, IL-6, iNOS, and TNF-α) in rat and primary microglia models of acute and chronic brain neuroinflammation. Therefore, antagonizing A2aR can be used as a treatment strategy for neonatal brain injury (Figure 1).

In short, caffeine can improve dysfunction in the central nervous system, which may be related to its anti-ARs effect.

### Neuroprotective Mechanism of Caffeine and the Inhibitory Effect on Phosphodiesterase

Caffeine is an inhibitor of phosphodiesterase. This can prevent the breakdown of cAMP. An increase in cAMP availability can stimulate the central nervous system. In addition, cAMP can stimulate lipolysis by triggering the activity of hormone sensitive lipase (HSL), which plays a crucial role in the adrenaline cascade (74). It also activates protein kinase A to phosphorylate several enzymes involved in the glucose and lipid metabolisms (75). Increased lipolysis leads to decreased dependence on glycogen use (76). Caffeine switches the substrate preference from glycogen to lipids by stimulating HSL activity and by inhibiting glycogen phosphorylase activity (77) and increases fatty acid oxidation. However, as a weak phosphodiesterase inhibitor, caffeine exerts its effect at much higher concentrations, and it is unlikely that caffeine mediates this effect at standard doses in newborns (17).

### Neuroprotective Mechanism of Caffeine and the Regulation of Intracellular Calcium

Caffeine can bind to calcium channels to release calcium from cells, while inhibiting voltage-sensitive calcium channels and thereby inhibiting neurotransmission. However, the dosage required for these effects may be at a toxic level. In addition, A1R can mobilize intracellular calcium (Ca^2+^) transmission through the inositol triphosphate receptor (IP3R) (78). Under physiological conditions, sleep deprivation induces forebrain basal cholinergic release to stimulate A1R, leading to IP3R activation and transcription factor changes (78, 79). Under pathological conditions such as hypoxia, abnormal adenosine increase may lead to excessive activation of A1R and a subsequent imbalance of IP3R and Ca^2+^ signals. Excessive Ca^2+^ release can directly lead to Ca^2+^ overload during hypoxia (80, 81) and is considered another important mechanism affecting the activity of astrocytes, microglia, and oligodendrocytes. Early studies reported that A1R reduces Ca^2+^ by inhibiting voltage-gated Ca^2+^ channels in excitable cells, including motor neurons (82) and retinal ganglion cells (83). This effect substantially inhibits the release of excitatory neurotransmitters such as glutamate during hypoxia. However, the latest study showed that A1R promotes Ca^2+^ overload during oligodendrocyte formation in a chronic hypoxia-induced periventricular white matter-injury model (59), which is considered an important mechanism of hypoxic injury due to poor differentiation of oligodendrocytes and impaired myelination (16). Caffeine can promote the differentiation and maturation of hypoxic oligodendrocytes by regulating the balance of Ca^2+^, thereby exerting a protective effect in neonatal hypoxic injury. A recent study reported that caffeine can regulate the intracellular Ca^2+^ of oligodendrocytes during hypoxia by antagonizing A1R and maintain intracellular Ca^2+^ homeostasis. Therefore, caffeine can inhibit adenosine-induced Ca^2+^ activity and prevent calcium overload damage (59) (Figure 1).

### Neuroprotective Mechanism of Caffeine and Interference With the GABA Receptor

In addition to antagonizing AR, inhibiting phosphodiesterase, and promoting intracellular calcium release, caffeine also has other biological effects, such as interfering with the GABA-A receptor and changing GABA transport (19, 48, 49). Conversely, caffeine affects the release of various neurotransmitters by blocking ARs, such as norepinephrine, dopamine, acetylcholine, serotonin, glutamate, and GABA (17). Other studies have shown that the migration and entry of GABA neurons into the hippocampal circuits of caffeine-administered mouse offspring during pregnancy and lactation are delayed in the 1st week after birth. The adult offspring of these mice showed decreased GABA neurons, increased excitability of the neural network, susceptibility to epilepsy, and some cognitive deficits (32). These effects suggest that rodents exposed to caffeine during pregnancy and lactation may produce offspring with neurodevelopmental deficits. Animal models of fetal drug exposure to caffeine have consistently revealed impaired GABA neurodevelopment (84).

However, the inhibition of phosphodiesterase requires a 20 times higher concentration of caffeine, blocking the GABA receptor requires a 40 times higher concentration, and Ca^2+^ release requires a 100 times higher concentration (85, 86). Therefore, these caffeine effects are rarely discussed in more detail.

## Use of Caffeine for Its Neuroprotective Effect in Prematurity

The neuroprotective strategies for premature infants with encephalopathy include administration of neuroprotective and functional recovery agents. Caffeine is considered to have direct neuroprotective effects in rodents (57) and direct and indirect neuroprotective effects in preterm infants, including decreasing apnea, reducing brain damage, and promoting brain-function recovery. Therefore, we believe that caffeine can exert neuroprotective effects in premature infants and that it is a candidate drug for the promotion of encephalopathy recovery in this patient population.

### Effect of Caffeine on the Neurodevelopment of Rodents

A vast number of animal studies support that caffeine has a direct neuroprotective effect on the developing brain (57, 87, 88). Caffeine may improve brain damage after hypoxia-ischemia by reducing nerve-cell apoptosis and exert a protective effect on the brain. Bona et al. (89) added low-dose caffeine (0.3 g/L) to the drinking water of neonatal rats in the 1st week of life (P1–P7) and found that caffeine can reduce neuronal necrosis and reduce brain hypoxic damage. In addition, studies have found that caffeine can affect neurodevelopment in newborn rats. Interestingly, exposure to a high dose of caffeine citrate (50 mg/kg/day) from the 1st day (P1) to the 12th day after birth can increase the prefrontal cortex volume. The total length and tree-like structure of the dendrites of pyramidal neurons is increased in the third layer of the cortex at P35, and this effect can continue until puberty (90). Alexander et al. (91) demonstrated that administering a single dose of caffeine (10 mg/kg/day) to rats with hypoxic-ischemic brain injury can reduce damage to the ligation side of the cortex and reduce the obvious expansion of the ventricular volume. The most recent study reported that, at clinically relevant doses, caffeine can promote primary neuron survival after hypoxic injury, and inhibit hypoxia-induced HIF-1α in primary cultures (100 μm caffeine) and in newborn mouse pups (63).

Caffeine also promotes the development of immature brain tissue and helps improve long-term behavior. Back et al. (16) exposed neonatal mice to hypoxia, causing changes in brain tissue volume, secondary ventricular dilatation, and reduction in myelin basic protein expression, and then administered caffeine (300 mg/L), which improved the formation of myelin, increased the proportion of immature oligodendrocytes, and reduced ventricle enlargement. Thomson et al. (92) also conducted animal experiments in premature baboons to further confirm that caffeine facilitated the development of immature brain tissue. A study by Kumral et al. (93) showed that methylxanthines (10 mg/kg/day) can improve the performance of neonatal rats with hypoxic-ischemic brain damage in the Morris water maze task, and improve the long-term learning and memory ability of rats.

These studies indicate that caffeine treatment is beneficial to the structural and functional recovery of perinatal hypoxic-ischemic brain injury. Furthermore, the latest research suggests that the neuroprotective effect of caffeine on the developing brain may be related to its anti-oxidation and anti-neuroinflammation effects (94, 95). A recent study showed that caffeine administered to newborn mice under normoxia can produce neuroprotective effects even at high doses (first dose 80 mg/kg; maintenance 20 mg/kg/day for 14 days), manifesting as decreased oxidative stress, myelin hyperplasia, and increased Golgi apparatus (96). However, a retrospective study showed that although caffeine is beneficial to the respiratory system of premature infants, it may have adverse molecular and cellular effects on the developing brain (68). During a brain growth spurt period (P2–P6), the risk of AR-related behavioral dysfunction, such as hyperalgesia and gait disorder, is significantly increased in neonatal SD rats treated with caffeine (15–20 mg/kg/day) by gavage (97). A risk of adenosine receptor-related behavioral dysfunction may exist in preterm newborns treated with caffeine for apnea. However, these negative results may be related to the test species, caffeine dose used, neurodevelopmental stage at the time of administration, and exposure time. In addition, animal models cannot fully explain the interaction between caffeine and other treatments in the neonatal intensive care unit that affect neurodevelopmental outcomes (68).

### Effect of Caffeine on the Neurodevelopment of Premature Infants

#### Potential Direct Effect

Clinical studies have also confirmed that caffeine has a protective effect on the development of the nervous system of premature infants and can promote functional recovery after injury. Although the CAP trial did not assess the direct effects of caffeine on neurodevelopment, reduction of AOP-related events and of intermittent hypoxia can help improve neurodevelopment (43, 44, 98, 99), suggesting that caffeine has a positive effect on neurodevelopment. The CAP trial found that caffeine treatment in premature infants with apnea can confer neurodevelopmental benefits (cognitive delay and cerebral palsy) in the early stages of development (27) but also has a beneficial impact on long-term neurological development. In a long-term study conducted by Schmidt et al. early administration of caffeine to premature infants with apnea improved the survival rates and decreased the incidence of cerebral palsy and cognitive delay during a 18- to 21-month follow-up period (10). Although the neurodevelopmental advantage was not statistically significant at 5 years of age (100), during follow-up at 11–12 years, motor impairment was reduced in relation to caffeine treatment (101). These results reflect the potential long-term neurodevelopmental benefits of caffeine in preterm infants (102).

Two studies using magnetic resonance imaging to evaluate the effects of early preventive caffeine treatment on white matter development in preterm infants found that caffeine can reduce white matter damage and improve white matter development. The researchers found that caffeine exposure was associated with decrease in the apparent diffusion coefficient (ADC) and a decrease in both axial and lateral ADCs representing more mature white matter tissue. ADC changes are related to caffeine-induced improvement in white matter microstructure development in premature infants (103, 104). Therefore, this suggests that caffeine has a direct neuroprotective effect other than the indirect effect of respiratory stimulation. A study on white matter injury in very preterm infants during the perinatal period showed beneficial effects of longer caffeine treatment at term-corrected age (105). For premature infants with birth weight ≤1,250 g, early administration of caffeine citrate can improve and correct the microstructure of white matter, but at the age of 11 years, when brain volume and white matter microstructure were compared to those of the placebo group, there was almost no difference. This shows that although caffeine may have a long-term effect on the development of the corpus callosum, any benefit to the brain structure of premature infants will diminish over time (106).

A prospective study by Hassanein et al. (107) revealed that in preterm infants younger than 34 weeks who were administered intravenous caffeine (20 mg/kg) after birth, cerebral cortex activity (conventional and amplitude-integrated electroencephalography) was increased at 36 weeks after corrected gestational age, but this did not increase seizure activity. In addition, studies by Doyle et al. (108) showed that premature infants who received early caffeine treatment for apnea had a significantly lower incidence of developmental coordination disorder at the age of 5 years, which may be another important benefit of caffeine. In addition, a studies by Maitre et al. showed that the use of caffeine (above the recommended daily doses of 5–10 mg/kg/day) can improve the audiovisual function of premature infants (109). A recent study showed that caffeine can increase the extraction of oxygen and have a short-term stimulating effect on brain metabolism; but, it does not reduce cerebral blood flow or affect brain activity (110).

#### Potential Indirect Effect

Long term mechanical ventilation itself is a strong risk factor for adverse neurodevelopmental outcome (cerebral palsy and a Bayley scale cognitive score for infant development of <85) at 18 months of age (111). In the CAP trial, infants with a shorter duration of positive pressure ventilation days had less dyskinesia (101). In very preterm infants who survived to 36 weeks of corrected gestational age, prolonged hypoxemia episodes in the first 2–3 months after birth were associated with adverse outcomes at 18 months (112). Prolonged caffeine treatment can reduce recurrent hypoxemia events in these preterm infants (44). Studies have shown that administering caffeine within 48–72 h after birth can reduce the occurrence of physiological patent ductus arteriosus (31, 113) and the demand for surgery (9). Therefore, caffeine can normalize cerebral blood flow by stabilizing the fluctuation of systemic blood pressure, thus conferring a neuroprotective effect in premature infants. In conclusion, the beneficial effects of caffeine on cardiopulmonary physiology in stabilizing systemic and cerebral hemodynamics and its ability to alleviate hypoxic respiratory depression may play an indirect role in neuroprotection.

## Conclusion

This article reviewed the data available on the effects of caffeine on encephalopathy in prematurity. Clinical studies have shown that caffeine has a beneficial effect on the immature brain; this effect depends on the age at start of administration, the regular dose of caffeine administered, the neurodevelopmental stage at the time of administration, and the duration of exposure. Caffeine has a beneficial effect on the premature infant. Although animal experiments have suggested that higher doses of caffeine may be more beneficial, these results should still be cautiously considered. Therefore, in future preclinical studies, more attention must be paid to assessing the effects of different doses of caffeine on the structure and function of the developing brain and to determine the maximum dose and the best administration window for neuroprotection in premature infants. Regarding the mechanism of action of caffeine, the current focus is on its antagonism of ARs. Few studies have examined the exact molecular and cellular mechanisms of caffeine in preventing and treating encephalopathy in premature infants, and some neuroprotective effects cannot be explained by changes in A1 and A2a receptors alone. Whether caffeine has other mechanisms of action that aid in the neuroprotective effect of encephalopathy in premature infants requires further research and discussion.

## Author Contributions

The preliminary draft of the review was prepared by LY, XY, YZ, and NL. XX and JF critically revised the manuscript for intellectual content. All authors contributed to the article and approved the submitted version.

## Funding

This study was supported by Key R&D Guidance Plan Projects In Liaoning Province (2020JH1/10300001).

## Conflict of Interest

The authors declare that the research was conducted in the absence of any commercial or financial relationships that could be construed as a potential conflict of interest.

## Publisher's Note

All claims expressed in this article are solely those of the authors and do not necessarily represent those of their affiliated organizations, or those of the publisher, the editors and the reviewers. Any product that may be evaluated in this article, or claim that may be made by its manufacturer, is not guaranteed or endorsed by the publisher.
